# Thirty years of vaccination in Vietnam: Impact and cost-effectiveness of the national Expanded Programme on Immunization

**DOI:** 10.1016/j.vaccine.2014.12.017

**Published:** 2015-05-07

**Authors:** Mark Jit, Dang Thi Thanh Huyen, Ingrid Friberg, Hoang Van Minh, Pham Huy Tuan Kiet, Neff Walker, Nguyen Van Cuong, Tran Nhu Duong, Kohei Toda, Raymond Hutubessy, Kimberley Fox, Nguyen Tran Hien

**Affiliations:** a Department of Infectious Disease Epidemiology, London School of Hygiene and Tropical Medicine, London, UK; b Modelling and Economics Unit, Public Health England, London, UK; c National Institute of Hygiene and Epidemiology, Hanoi, Vietnam; d John Hopkins Bloomberg School of Public Health, John Hopkins University, Baltimore, MD, USA; e Center for Health System Research, Hanoi Medical University, Hanoi, Vietnam; f World Health Organization Representative Office for Viet Nam, Hanoi, Vietnam; g World Health Organization, Geneva, Switzerland; h World Health Organization Regional Office for the Western Pacific, Manila, Philippines

**Keywords:** DALY, disability adjusted life year, DPT, diphtheria–pertussis–tetanus vaccine, EPI, Expanded Programme on Immunization, LiST, Lives Saved Tool, Cost-effectiveness, Diphtheria, Immunization, Measles, Pertussis, Polio

## Abstract

**Introduction:**

Countries like Vietnam transitioning to middle-income status increasingly bear the cost of both existing and new vaccines. However, the impact and cost-effectiveness of the Expanded Programme on Immunization (EPI) as a whole has never been assessed on a country level.

**Methods:**

Data on vaccine-preventable disease incidence and mortality from Vietnam's national surveillance was analysed to estimate the likely impact that vaccination in 1980–2010 may have had. Adjustment for under-reporting was made by examining trends in reported mumps incidence and in case-fatality risks for each disease. The same data were separately analysed using the Lives Saved Tool (LiST) to give an alternative estimate of impact. The financial cost of EPI in 1996–2010 was also estimated from the perspective of service provider.

**Results:**

National surveillance data suggests that up to 5.7 million diseases cases and 26,000 deaths may have been prevented by EPI. Analysis using LiST suggests that even more deaths (370,000) may have been prevented by measles and pertussis vaccination alone. The cost-effectiveness of EPI is estimated to be around $1000–$27,000 per death prevented.

**Conclusion:**

Two separate approaches to assessing EPI impact in Vietnam give different quantitative results but a common conclusion: that EPI has made a substantial impact on mortality and represents good value for money.

## Highlights

• Vietnam's EPI has caused sharp decreases in vaccine-preventable disease incidence.
• EPI may have saved over 370,000 lives.
• EPI represents good value for money.

## 1 Introduction

The Expanded Programme on Immunization (EPI) was established by the World Health Organization (WHO) in 1974 to support countries in increasing uptake of vaccines against measles, diphtheria, pertussis, tetanus, poliomyelitis and tuberculosis. Between 1980 and 2011, global coverage of the third dose of diphtheria–pertussis–tetanus vaccine (DPT) increased from 20% to 83%, while that of measles-containing vaccine increased from 16% to 85% [1].

A World Bank report [2] considered that vaccines covered by EPI are among the most cost-effective interventions available, with measles immunization estimated to cost $10 per disability life year (DALY) prevented, and DPT immunization $25 per DALY prevented. A more recent analysis [3] suggested that the incremental cost per death averted of EPI ranges from $274 in South Asia to $1754 in Europe and Central Asia in 2001 US$, and is about $478 in East Asia and the Pacific. However, the impact and cost-effectiveness of the EPI vaccine package has never been formally evaluated on a national level. Hence previous cost-effectiveness analyses have relied on extrapolation of limited data to a global level.

A key indicator of EPI success is observed reductions in vaccine-preventable disease incidence and mortality [4]. However, passive surveillance of trends in disease incidence may be affected by underreporting, particularly of cases that do not present for health care. Furthermore, the degree of underreporting may change over time as case definitions, access to care and surveillance systems evolve. On the other hand, active surveillance methods such as cross-sectional surveys without major sources of bias are normally too resource-intensive to be conducted regularly in low and middle income countries. For mortality, establishing the cause of death is difficult when symptoms are non-specific and children have multiple co-morbidities at the time of death. Lastly, even if declines in severe disease and mortality are well-documented, these may be due to improved access to care, nutrition and general health as well as vaccination.

EPI was first introduced in Vietnam in 1981, and became one of six national targeted health programmes in 1985. The programme originally vaccinated infants against six diseases (diphtheria, tetanus, pertussis, poliomyelitis, measles, and tuberculosis). By 2009, 96% of children less than 1 year old were recorded as having received three doses of DPT. EPI's successes include elimination of polio in 2000 and of maternal and neonatal tetanus in 2005.

Vietnam has benefitted from Gavi support for vaccine introduction and health systems strengthening, but eligibility for these funds may end as Vietnam transitions to middle-income status. Hence both the current EPI as well as new vaccine introductions will be increasingly funded by national resources. Yet there are competing priorities for Vietnam's public investments both within and outside the health sector. As the government plans for future investments, it is critical to understand the impact and value of Vietnam's EPI.

The goal of this study is to assess the impact and cost-effectiveness of Vietnam's EPI in reducing mortality and morbidity associated with vaccine-preventable diseases over its 30 year history. The study addresses a key evidence gap in providing the first national impact and economic evaluation of EPI. To do so, two complementary methods were used. First, data on vaccine-preventable disease incidence and mortality from national surveillance were analysed to estimate the likely impact that 30 years of vaccination may have had. Second, the Lives Saved Tool (LiST) was used. LiST is a model to estimate the number of lives saved by different packages and coverage levels of health interventions by combining evidence about the effectiveness of maternal, neonatal and child health interventions with country specific information about cause of death and current coverage of health interventions.

## 2 Methods

### 2.1 Statistical modelling based on surveillance data

The National Institute of Hygiene and Epidemiology records notifications of cases and deaths attributed to measles, diphtheria, pertussis, tetanus and polio in 1980–2010 in all ages. Surveillance was based on clinical, epidemiological and microbiological confirmation for polio (since 1992) and measles (since 2000), and clinical diagnosis only for other diseases. Case definitions are given in Appendix A.1; these were unchanged over the entire period 1980–2010, apart from a change in the measles case definition in 2003 by which time measles incidence had reached very low levels.

Decreases in notified cases give an indication of the impact of vaccination. However, incidence may have declined due to reasons unrelated to vaccination, such as changes in case ascertainment and disease risk factors. To control for non-vaccine related changes, annual vaccine-preventable disease notifications were adjusted based on changes in mumps incidence in the same year (see Appendix A.3). Mumps was chosen as a control variable because there is no mumps vaccination programme currently in place in Vietnam, so any changes in reported mumps incidence must be due to non-vaccine related causes. A regression curve was fitted to annual mumps incidence to smooth out year-to-year variations (see Appendix A.2).

The National Institute of Hygiene and Epidemiology records administrative coverage for measles, DPT and polio vaccines in 1980–2010, except for 1988. Reported incidence for measles, diphtheria, pertussis and polio was related to routine dose coverage in order to investigate the temporal association between increasing vaccine coverage and decreasing disease incidence (hence providing evidence that vaccination is a cause of disease decline). Tetanus was not modelled since reductions in neonatal tetanus incidence are not easily associated with infant DPT vaccination alone, and are due also to maternal vaccination. For parsimony, coverage of catch-up, second dose and booster programmes was not considered; these programmes took place in the latter years of the period 1980–2010 when disease incidence had already reached fairly low levels. Twelve linear regression models were used to explore the association between incidence (with or without the mumps adjustment factor) and vaccine coverage over the past three years (see Table 1). The reduction in disease incidence attributed to vaccination in a particular year was assumed to be equal to the difference between incidence in that year estimated by each model (unless this was negative, in which case it was rounded to zero) and incidence predicted to occur by the same model when vaccine coverage was 0%. Models giving the largest and smallest estimated number of vaccine-prevented deaths were selected to provide an uncertainty range. If notification data alone or mumps-adjusted notification data (without regression modelling) gave the largest or smallest number this was selected instead.

The number of deaths attributed to vaccine-preventable diseases may have declined due to reasons unrelated to vaccination, such as improved healthcare and nutrition. To adjust for this, it was assumed that reductions in deaths due to reasons unrelated to vaccination reduced the case-fatality risk of disease without affecting disease incidence. On the other hand, vaccination is assumed to simply prevent disease from occurring in the first place, rather than to reduce the severity (and hence risk of death) of cases. Hence the number of deaths prevented by vaccination in a particular year was assumed to be the estimated number of disease cases prevented by vaccination in that year (as described above), multiplied by the case-fatality risk for the disease in that year estimated using national surveillance data (see Appendix A.5 for equations).

### 2.2 Estimation of the number of deaths prevented using the Lives Saved Tool (LiST)

LiST was used to model under-five mortality due to measles and pertussis in 1980–2010. Polio and diphtheria have limited roles in under-five mortality while neonatal pertussis is not directly prevented by infant vaccination alone. Details of LiST methodology have been published elsewhere [5]. Briefly, a complete LiST projection was constructed for 2000 using all coverage data, health status information and mortality rates. The 1980 coverage and health status rates were entered for the years 2001–2005 in order to extrapolate the most likely proportionate cause of death in neonates and 1–59 months olds using a method previously reported in the literature [6]. These values were then used in the baseline LiST projection for the years 1980–2010. Full details of data sources and methodology are given in Appendix A.6.

The LiST analysis was compared to the reduction over time in the number of cases of and deaths due to measles and pertussis (in 1980–2010) according to national surveillance data. To do this, the average annual number of cases and deaths between 1980 and 1986 was calculated and compared to the average annual value between 2000 and 2010, for both measles and pertussis, to calculate a percent reduction over time.

### 2.3 EPI costs

The financial cost of Vietnam's EPI in 1996–2010 was estimated from the perspective of the service provider. Records prior to 1996 (including start-up costs) were not available. All the inputs (ingredient) used in implementing the programme were captured. The cost per vaccine dose was estimated by incorporating all relevant ingredients, such as personnel, supplies, vaccine procurement, operations and logistics (see Appendix A.7). The total annual cost of each vaccine was estimated by multiplying the average cost per dose by the total number of doses used by the program in that year. Costs were presented in 2010 US$.

## 3 Results

Models relating notifications incidence and vaccine coverage for each of the four diseases appear to fit data well, based on visual inspection and by comparing their Akaike Information Criterion (see Appendix A.4 for results). They capture initial high disease incidence prior to vaccination as well as its rapid decline as EPI was rolled out nationally. Coefficients relating disease notifications to vaccine coverage were negative (including the entire 95% confidence interval; data not shown), supporting the hypothesis that vaccination has been a cause of disease decline. Models predict a spike in diphtheria and pertussis incidence around 2002 which did not occur in practice, despite a sharp decline in DPT coverage from 96% in 2001 to 75% in 2002. The reason for this may be herd protection from existing vaccinated cohorts; these indirect effects are poorly captured by a linear model [7]. The best fitting model was model 11 for polio, and model 9 for all other diseases.

**Fig. 1:**
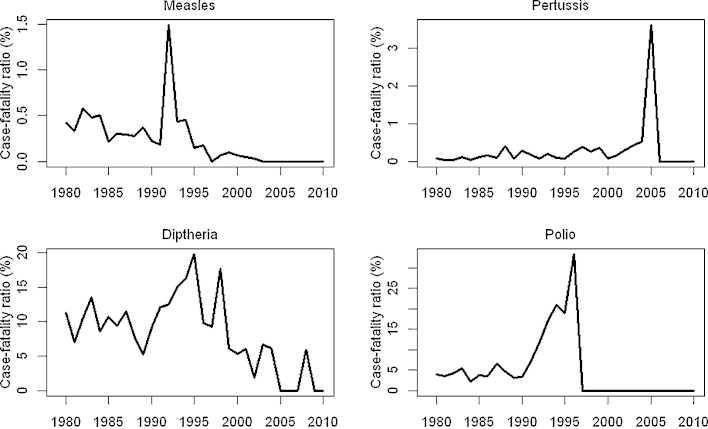
Case-fatality risk of measles, diphtheria, pertussis and polio from 1980 to 2010, based on notified cases and deaths. The case-fatality risk for polio after 1996 could not be calculated as polio had been eliminated by then.

**Fig. 2:**
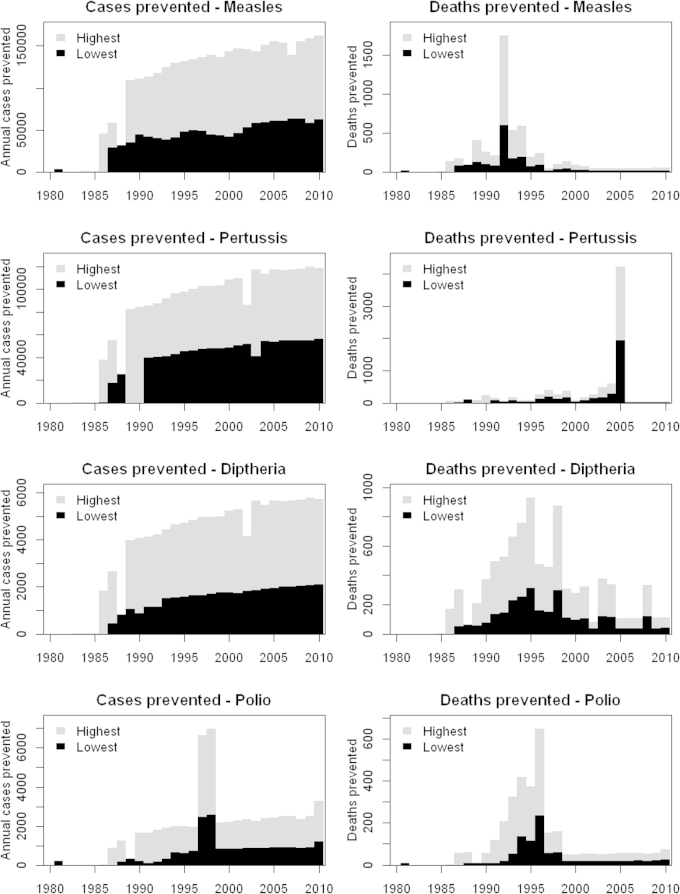
Highest and lowest numbers of disease cases and deaths prevented by EPI from 1980 to 2010 based on models fitted to national surveillance data.

Case-fatality risks for measles, pertussis, diphtheria and polio declined in 1980–2010 (Fig. 1). Temporary increases in 1993 (measles), 2005 (pertussis) and 1990–1996 (polio) may represent outbreaks in socioeconomically disadvantaged areas with a higher case-fatality risk, due to lower vaccine coverage in these geographically restricted regions. High reported case-fatality risk for polio in the decade prior to elimination may reflect improved ascertainment of polio deaths due to the attention being given to this disease. The annual number of disease cases prevented by vaccination has increased since 1980, due to both improved vaccine coverage and increasing birth cohort size (Fig. 2). However, the number of deaths prevented has decreased, because of declining case-fatality risks, particularly for measles. In total, 2.3–5.7 million diseases cases and 10,000–26,000 deaths are estimated to have been prevented by EPI in 1980–2010. The largest impact (in terms of deaths prevented by vaccination) was obtained when using model 12 for pertussis, and using unadjusted surveillance data alone for other diseases. The least impact was obtained when using model 5 for polio, and model 1 for other diseases.

LiST estimates even greater benefit from EPI. According to LiST, about 370,000 under-five deaths may have been prevented by two EPI vaccines (measles and pertussis), primarily through prevention of measles mortality. This is mainly because LiST estimates of mortality for measles (based on WHO estimates) are about 70–200 times higher than those estimated from adjusted surveillance data, although estimates for pertussis are similar. However, the proportionate reduction in the number of deaths between 1980 and 2010 is similar: 99–100% and 97–100% for measles and pertussis using national data, compared to 96% and 83% using LiST. LiST suggests that EPI may have been responsible for around 15% of under-five mortality decline in Vietnam since 1980, mainly due to measles vaccination. Results for all four diseases are shown in Table 2, together with corresponding figures using LiST.

The cost of EPI over 15 years (1996–2010) is estimated at $154.5 million, consisting of $41.8 million for routine DPT, $28.3 million for polio, $8.6 million for first measles dose, $5.41 million for second measles dose, $0.25 million for DPT campaigns, $46.8 million for polio campaigns and $23.4 million for measles campaigns (see Appendix A.7 for costs disaggregated by year). Using the lowest estimate of the number of deaths prevented in 1996–2010 (9400 deaths), EPI cost $27,000 per life saved. Since costs are only available for half time period 1980–2010 over which deaths prevented are estimated, we use half the much higher estimate of deaths prevented in 1980–2010 according to LiST (half of 370,000 deaths). This suggests that EPI only cost $1000 per life saved. Indeed EPI may be even more cost-effective than suggested here, and may even be net cost-saving, as neither estimate incorporates cost savings to the health sector due to reduced treatment. Furthermore, almost half the cost of EPI was spent on polio vaccination, whose main aim is not mortality prevention.

## 4 Discussion

We have used two methods to assess the impact of EPI on disease incidence and mortality in Vietnam since 1980. Statistical analysis of national surveillance data suggests that up to 5.7 million disease cases and 26,000 deaths may have been prevented by EPI. A significant temporal association between disease incidence and vaccine coverage was found. Using LiST suggests that about 370,000 deaths may have been prevented by two EPI vaccines (measles and pertussis). Cost-effectiveness analysis using financial data suggests that EPI costs around $1000–$27,000 per death prevented.

The lower end of the range for cost-effectiveness, based on LiST estimates of mortality, is close to a Disease Control Priorities Network estimate for East Asia and the Pacific of EPI costing $434 per death prevented [3]. This is unsurprising since both LiST and the Disease Control Priorities Network estimate vaccine-preventable mortality based on WHO cause-specific mortality figures. However, the cost-effectiveness ratio estimated using national surveillance data is much greater. This is because LiST estimates more than ten times the number of measles deaths prevented by EPI compared to national surveillance data, although the proportionate reduction in deaths is much more similar between the two approaches.

The difference in absolute numbers may stem from underascertainment of measles deaths in national surveillance data due to either misattribution of measles deaths to other causes, or measles patients not seeking health care. Furthermore, if underascertainment has decreased since 1980 (as may be expected due to improved surveillance and health care access) then the magnitude of decline in measles deaths from 1980 to 2010 will be underestimated.

To adjust for underascertainment, we use mumps notifications as a control variable to represent changes in infectious disease notifications that are not related to vaccination. Mumps-containing vaccines are not routinely administered in Vietnam; hence any changes in notifications of mumps cases must be due to the other factors, which are assumed to equally affect other communicable diseases. The control variable accounts for both (i) improvements in disease ascertainment which may have caused incidence to rise, and (ii) changes in disease risk factors (such as improved hygiene) which may have caused incidence to fall. There was a decreasing trend in mumps incidence between 1980 and around 1990 (see Appendix A.2), suggesting that declines in disease risk factors outweighed the effect of improved ascertainment until the latter part of the study period. This means that mumps-adjusted disease incidence is higher than unadjusted incidence in our model for the first part of the study period. Unfortunately, it is not possible to disentangle the individual effects of both changes through a single control variable. Another limitation is that this method would not have captured changes in diphtheria, pertussis, measles or polio ascertainment that did not affect mumps. For example, case ascertainment for measles may have improved more than for mumps, due to the regional focus on measles elimination [8].

Several studies have examined underascertainment in Vietnamese surveillance sources. A household study in Bavi District compared mortality recorded through the Commune Population Registration System (CPRS) in 1999–2000 with that estimated using three other methods (re-census, communal death registration and neighbourhood surveys) [9]. This suggested that the CPRS may have missed around 19% of deaths, particularly in infant and the elderly, but did not examine deaths that may have been assigned to the wrong cause. A cross-sectional survey in 2008/2009 comparing surveillance case reports with cases recorded in health care logbooks of commune health stations found a shortfall of 61.3%, 47.5% and 56.3% for influenza-like illness, pneumonia and severe pneumonia, respectively [10]. Our own estimates using mumps as a control variable suggests that in 1999, 48% more mumps cases were missed compared to 2010.

A second potential cause of the difference between the analysis of surveillance data and LiST is that the former analysis adjusts for reductions in incidence and mortality that may have occurred due to non-vaccine related factors such as health care access, population structure, overall health, crowding and sanitation. Reduction in infectious disease incidence due to non-vaccine causes is captured using the adjustment factor based on mumps notifications. Indeed, the incidence of mumps notifications decreased between 1980 and 1990, possibly due to improvements in health, before increasing after 1990, possibly due to improved surveillance. Reduction in mortality due to non-vaccine causes is captured by assuming that case-fatality risks in a particular year would still hold in the absence of these non-vaccine factors, even if vaccination had not taken place, and vaccination would only reduce the number of cases of the disease.

One limitation of our statistical analysis of surveillance data is that it relates vaccine coverage to disease incidence using linear regression models that do not capture non-linear effects such as herd protection [7]. The models adequately describe the overall pattern of disease decline as coverage increases from zero to close to 100% since herd effects are minimal at both extremes of the coverage range. However, outbreaks due to short-term fluctuations in coverage are less well captured.

LiST takes a different approach. LiST is a multi-cause model of mortality, which captures the interactions between different interventions that prevent or allow deaths to occur. This relies on WHO estimates of mortality using natural history models [11]. However, LiST assigns mortality reductions due to preventive interventions (such as vaccination) before therapeutic interventions (such as nutritional supplementation and improved access to hospital care). For instance, the impact of measles vaccination is applied to measles mortality first, and the effect of vitamin A therapy on measles mortality is only applied to the measles deaths that remain after the application of vaccination. Consequently, LiST is likely to assign a greater proportion of the decline in measles mortality since 1980 to vaccination compared to the analysis of surveillance data. Hence the difference in the two model estimates may be due to under-ascertainment in national data, overestimation by LiST, or a combination of both.

These methodologies can be used in other settings with vaccine-preventable disease surveillance to retrospectively estimate the impact of EPI. The regression method can be used in any setting with (i) an adequate time series of case and death notifications, and (ii) vaccine coverage for both diseases prevented by EPI vaccines as well as another disease (such as mumps) not affected by existing vaccines. LiST has also been used in other settings. For example, one analysis showed that 11% of recent child mortality reduction in Niger is likely due to vaccines [12]. LiST has also been used to prospectively estimate the potential impact of introducing vaccines or accelerating broader vaccine introduction [13], [14].

The difference in deaths averted between the two methods in our analysis is very large, even after adjusting surveillance data for under-ascertainment. This highlights the importance of using multiple methods to estimate vaccine impact when directly observed and actively reported data are not available, as is the case in most low and middle income countries. Having multiple estimates allows triangulation of a likely range in which the true value of deaths averted is likely to lie. However, further investigation into the extent of under-ascertainment in surveillance data and the importance of non-vaccine causes of mortality decline is needed in order to determine which of the two estimates is closest to the truth.

Despite these differences, it is highly reassuring that the broad conclusion from both approaches is the same: Vietnam's EPI has made a substantial impact on mortality and is very likely to be cost-effective, even under conservative assumptions. As more countries graduate from Gavi funding and rely on internal resources, impact and economic analyses of national vaccination programmes such as EPI are likely to become increasingly important. Furthermore, our methodology suggests that both surveillance and modelling play important and complementary roles in such estimates (Table 3).

## Conflict of interest statement

None declared.

## Figures and Tables

**Table 1:** Linear regression models used to relate disease incidence to vaccine coverage. Symbols: *x*_*i*_ = incidence of notified cases in year *i*, *y*_*i*_ = incidence of notified cases in year *i* adjusted using mumps data*, c*_*i*_ = vaccine coverage in year *i* (*c*_*i*_ = 0 for *i* < 1980).

Model number	Model equation	Dependent variable		Independent variable(s)		
Disease incidence *x*_*i*_	Mumps-adjusted disease incidence *y*_*i*_	Vaccine coverage in the same year *c*_*i*_	Vaccine coverage in the previous year *c*_*i−*1_	Vaccine coverage two years ago *c*_*i−*2_
1	*x*_*i*_ ∼ *c*_*i*_	✓		✓		
2	*x*_*i*_ ∼ *c*_*i*_ + *c*_*i−*1_	✓		✓	✓	
3	*x*_*i*_ ∼ *c*_*i*_ + *c*_*i−*1_ + *c*_*i−*2_	✓		✓	✓	✓
4	*x*_*i*_ ∼ *c*_*i*_ + *c*_*i−*2_	✓		✓		✓
5	*x*_*i*_ ∼ *c*_*i−*1_	✓			✓	
6	*x*_*i*_ ∼ *c*_*i−*1_ + *c*_*i−*2_	✓			✓	✓
7	*y*_*i*_ ∼ *c*_*i*_.		✓	✓		
8	*y*_*i*_ ∼ *c*_*i*_ + *c*_*i−*1_		✓	✓	✓	
9	*y*_*i*_ ∼ *c*_*i*_ + *c*_*i−*1_ + *c*_*i−*2_		✓	✓	✓	✓
10	*y*_*i*_ ∼ *c*_*i*_ + *c*_*i−*2_		✓	✓		✓
11	*y*_*i*_ ∼ *c*_*i−*1_		✓		✓	
12	*y*_*i*_ ∼ *c*_*i−*1_ + *c*_*i−*2_		✓		✓	✓

**Table 2:** Estimated EPI impact on cases and deaths due to measles, pertussis, diphtheria and polio.

	Measles		Pertussis		Diptheria		Polio		Total	
Low^1^	High^1^	Low^1^	High^1^	Low^1^	High^1^	Low^1^	High^1^	Low^1^	High^1^
Using national surveillance data										
Cases 1980	40,000	110,000	35000	78,000	1300	3900	580	1800	76,880	19,3700
Cases 2010	2900	15,000	0	6900	6	610	0	540	2906	23,050
Deaths 1980	170	460	26	59	150	440	22	70	368	1029
Deaths 2010	0.85	4.4	0	2.1	0.11	12	0	12	0.96	30.5
Deaths per 1000 cases 1980	4.2	4.2	0.76	0.76	110	110	38	38	0.0048	0.0053
Deaths per 1000 cases 2010	0.3	0.3	0.31	0.31	19	19	23	23	0.00033	0.0013
Vaccine prevented cases 1980–2010	1.2	3.1	1	2.4	0.038	0.11	0.019	0.058	2.257	5.668
Vaccine prevented deaths 1980–2010^2^	1900	5300	3700	8200	2800	8800	960	3200	9360	25,500
Vaccine prevented cases 1996–2010^3^	0.81	2.2	0.77	1.7	0.028	0.08	0.017	0.045	1.625	4.025
Vaccine prevented deaths 1996 - 2010^2,3^	380	1100	3300	7300	1500	4400	600	1600	5780	14,400
% reduction in cases due to vaccination (2010 vs. 1980)	93	86	100	91	100	85	100	71	96%	88%
% reduction in deaths due to vaccination (2010 vs. 1980)	99	99	100	96	100	97	100	82	87%	96%

Using LiST										
Deaths 1980	23,000		300						26,800	
Annual deaths 2000–2010	1000		50						1250	
Vaccine prevented deaths 1980–2010	366,000		5000						411,000	
% reduction in deaths due to vaccination (1980 to 2000–2010)	96%		83%						95%	

^1^ “Low” and “high” represent figures from the highest and lowest results (in terms of disease impact) of twelve linear regression models used.

^2^ Calculated as number of cases prevented by vaccination in each year × case-fatality risk in the same year.

^3^ Vaccine impact for 1996–2010 only was used to calculate cost-effectiveness, since financial costs were only available starting from 1996.

**Table 3:** Two approaches to retrospectively estimate the impact of a vaccination programme.

	Surveillance based estimation	Impact model based estimation
Description	Monitor cases and deaths due to a disease both before and during vaccination	Model the likely reduction in morbidity and/or mortality based on disease natural history and vaccine effectiveness
Strengths	Direct observation of changes in incidence. Hence able to capture complex nonlinear effects such as herd protection	Less affected by surveillance biases
Limitations	Affected by underascertainment or misattribution of disease/deaths, as well as changes in morbidity/mortality due to non-vaccine related causes	Estimated vaccine impact is dependent on the order in which interventions are applied when there are multiple interventions that can affect disease incidence and mortality (such as vaccination and treatment)
