# Supplementary material for: Thirty years of vaccination in Vietnam: Impact and cost-effectiveness of the national Expanded Programme on Immunization
Source: Vaccine. 2015 May 7;33(Suppl 1):A233–9. doi: 10.1016/j.vaccine.2014.12.017 (PMC4428532; doi:10.1016/j.vaccine.2014.12.017)
Supplement: Supplementary file 1 [file mmc1.docx]

**Supplementary Appendix**

**Contents**

A.1. Definitions used for case notifications by NIHE

A.2. Fitting a smoothing function to reported mumps incidence

A.3. Adjusting reported vaccine-preventable disease incidence

A.4. Linear models relating disease incidence to vaccine coverage

A.5. Calculating the number of cases and deaths prevented by vaccination

A.6. Estimation of vaccine-prevented measles and pertussis deaths using LiST

A.7. Details about estimates of EPI costs

**A.1. Definitions used for case notifications by NIHE**

| **Disease** | **Case definitions** | | | **Remarks** |
| --- | --- | --- | --- | --- |
|  | **Clinical case definition** | **Laboratory criteria for diagnosis** | **Case classification** |  |
| Measles | 1. Any person with fever AND maculopapular rash AND one of these symptoms: Cough, coryza (i.e. runny nose) OR conjunctivitis (i.e. red eyes); OR 2. Any person in whom a clinician suspects measles infection. | Presence of measles-specific IgM antibodies in the serum. | **Laboratory-confirmed:** A case that has a positive test with measles-specific IgM antibodies.  **Epidemiologically-confirmed:** A case without specimen that meets the clinical case definition and is linked epidemiologically to a laboratory-confirmed case or an epidemiologically-confirmed case.  **Clinically-confirmed:** A case without specimen that meets the clinical case definition  **Discarded:** A suspected case that does not meet the clinical or laboratory definition. | Before 2004, measles was mainly diagnosed by clinically. Measles case-based surveillance was rolled-out in 2002, with laboratory surveillance becoming widespread in 2004. |
| Diphtheria | A person with laryngitis OR pharyngitis OR tonsillitis AND the presence of a adherent membrance of the tonsils, pharynx, and/or nose. | Isolation of Corynebacterium diphtheria from specimen; OR a fourfold greater rise in serum antibody. | **Clinically-confirmed:** A case without specimen that meets the clinical case definition  **Laboratory-confirmed:** A case that is isolated Corynebacterium diphtheria from specimen or has a fourfold greater rise in serum antibody | No laboratories involved in diphtheria surveillance, but most reported cases tested with isolation in hospitals since 2002. |
| Pertussis | A person a cough lasting at least two weeks AND with at least one of the following symptoms: (1) Paroxysms of coughing, (2) Inspiratory whoop, (3) post-tussive vomiting | Isolation of Bordetella pertussis from naso-pharyngeal secretions OR positive paired serology |  |  |
| Polio | Any child under 15 years of age with acute flaccid paralysis (AFP); OR Any person of any age with paralytic illness if polio is suspected | Isolation of wild poliovirus | **Laboratory-confirmed:** An AFP case that is isolated wild poliovirus in one or both the stool specimens  **Clinically-confirmed:** An AFP case that has residual paralysis/weakness at 60 days OR death before follow-up OR lost to follow-up  **Discarded:** An AFP case that does not isolate wild poliovirus from two stool specimens or does not have residual paralysis/weakness at 60 days |  |
| Mumps | Acute onset of unilateral or bilateral tender, self-limited swelling of the parotid or other salivary gland, lasting two or more days and without other apparent cause | No | **Clinically-confirmed:** A case that meets the clinical case definition |  |

**A.2. Fitting a smoothing function to reported mumps incidence**

A Loess (locally weighted polynomial regression) curve was fitted to annual reported mumps incidence to smooth out year-to-year variations [1]. Fitting was done using the least squares method with degree of smoothness 1.5. The **loess** function in the computer package R (version 2.15.1) was used for this purpose. The figure below shows the Loess curve and the original mumps data. There was a decreasing trend in reported mumps incidence until 1990, followed by an increasing trend.


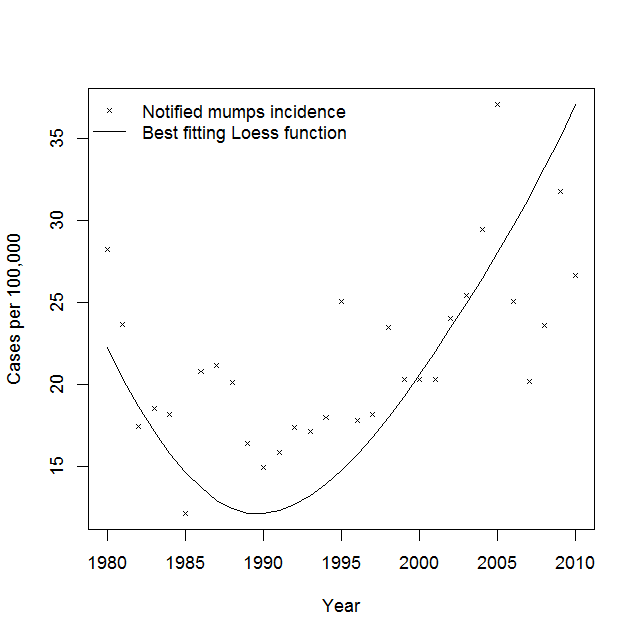


**A.3. Adjusting reported vaccine-preventable disease incidence**

Let

*x_i_* be the incidence of notified cases of disease D in year *i*,

*m_i_* be the incidence of notified cases of mumps in year *i*,

*y_i_* be the incidence of notified cases in year *i* adjusted using mumps data*,* and,

*c_i_* be vaccine coverage for disease D in year *i*.

Then *y_i_* = *x_i_* ⋅ *y_2010_*/*y_i_*

**A.4. Linear models relating disease incidence to vaccine coverage**

Twelve linear models were fitted to the data to relate disease incidence (either adjusted or unadjusted by mumps incidence) to vaccine coverage over the three most recent years:

| **Model #** | **Equation** | **Dependent variable** | | **Independent variable(s)** | | |
| --- | --- | --- | --- | --- | --- | --- |
|  |  | **Disease incidence** | **Mumps-adjusted disease incidence** | **Vaccine coverage in same year** | **Vaccine coverage in previous year** | **Vaccine coverage two years ago** |
| 1 | *y_i_* ~ *c_i_*. | ✓ |  | ✓ |  |  |
| 2 | *y_i_* ~ *c_i_* + *c_i-1_* | ✓ |  | ✓ | ✓ |  |
| 3 | *y_i_* ~ *c_i_* + *c_i-1_* + *c_i-2_* | ✓ |  | ✓ | ✓ | ✓ |
| 4 | *y_i_* ~ *c_i_* + *c_i-2_* | ✓ |  | ✓ |  | ✓ |
| 5 | *y_i_* ~ *c_i-1_* | ✓ |  |  | ✓ |  |
| 6 | *y_i_* ~ *c_i-1_* + *c_i-2_* | ✓ |  |  | ✓ | ✓ |
| 7 | *x_i_* ~ *c_i_*. |  | ✓ | ✓ |  |  |
| 8 | *x_i_* ~ *c_i_* + *c_i-1_* |  | ✓ | ✓ | ✓ |  |
| 9 | *x_i_* ~ *c_i_* + *c_i-1_* + *c_i-2_* |  | ✓ | ✓ | ✓ | ✓ |
| 10 | *x_i_* ~ *c_i_* + *c_i-2_* |  | ✓ | ✓ |  | ✓ |
| 11 | *x_i_* ~ *c_i-1_* |  | ✓ |  | ✓ |  |
| 12 | *x_i_* ~ *c_i-1_* + *c_i-2_* |  | ✓ |  | ✓ | ✓ |

Note that *c_i_* = 0 for *i* < 1980 in the above equations.

The results of the model fit as well as the goodness of fit (using the Akaike Information Criterion) for measles (as an illustration) is shown in the table below. The Akaike Information Criterion is a measure of the goodness of fit of the models, taking into account the number of degrees of freedom (explanatory variables). In this fit, all data points (notifications incidence and vaccine coverage in a particular year) are treated as having equal weight.

| **Model #** | **Goodness of fit parameters** | | | **Model coefficients** | | | | |
| --- | --- | --- | --- | --- | --- | --- | --- | --- |
|  | **Deviance** | **Df** | **Akaike Info. Criterion** | **Intercept** | **Cover-age t** | **Cover-age t-1** | **Cover-age t-2** | **Include?** |
| 1 | 4040000 | 2 | 445 | 2040 | -19.1 | 0 | 0 | 1 |
| 2 | 2750000 | 3 | 423 | 1940 | 8.27 | -26.8 | 0 | 0 |
| 3 | 2640000 | 4 | 410 | 1960 | 9 | -35.4 | 7.79 | 0 |
| 4 | 3570000 | 3 | 430 | 1980 | -9.43 | -9.42 | 0 | 1 |
| 5 | 2960000 | 2 | 436 | 1970 | -18.9 | 0 | 0 | 1 |
| 6 | 2840000 | 3 | 424 | 1990 | -27.1 | 8.07 | 0 | 0 |
| 7 | 479000 | 2 | 382 | 935 | -8.86 | 0 | 0 | 1 |
| 8 | 395000 | 3 | 366 | 908 | -1.79 | -6.91 | 0 | 1 |
| 9 | 351000 | 4 | 354 | 920 | -1.35 | -12.3 | 4.91 | 0 |
| 10 | 463000 | 3 | 371 | 926 | -7.45 | -1.35 | 0 | 1 |
| 11 | 453000 | 2 | 380 | 882 | -8.49 | 0 | 0 | 1 |
| 12 | 392000 | 3 | 366 | 902 | -14.4 | 5.85 | 0 | 0 |

The figure below shows annual incidence predicted by the models compared to observed incidence from surveillance data (with and without mumps adjustment):

*
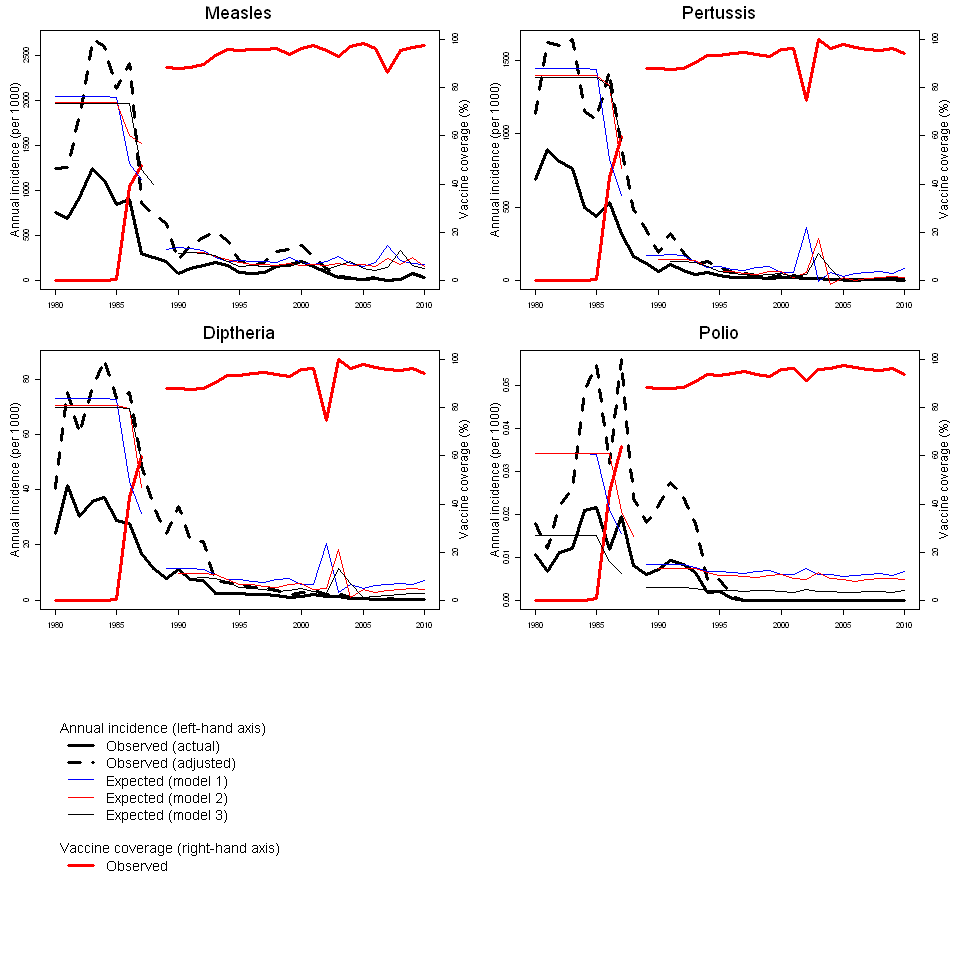
*

**A.5. Calculating the number of cases and deaths prevented by vaccination**

Let

*Y*(*c*) be incidence of disease D (in person-years) predicted by the best-fitting model (Model 1) given vaccine coverage *c* in the same year

*p_i_* be the population of Vietnam in year *i*

*z_i_* be the incidence of notified deaths due to disease D in year *i*

Then be the case-fatality risk of disease D in year *i, d_i =_ z_i_/x_i_.* Note that *x_i_* (incidence of notified cases) is used instead of *y_i_* (incidence of notified cases after multiplying by the mumps adjustment factor) in this equation, because we assume that the adjustment factor is the same for both cases and deaths due to disease D.

Furthermore:

- the estimated number of cases of disease D prevented by vaccination in year *i* is *Y*(0) *p_i_* – *Y*(*c_i_*) *p_i_*.
- the estimated number of deaths due to disease D that would have occurred in year *i* if vaccination had not occurred since 1980 is *Y*(*0*) *p_i_* *d*_i._
- the estimated number of deaths due to disease D that were prevented by vaccination alone in year *i* is [ *Y*(0) *p_i_* – *Y*(*c_i_*) *p_i_*] *d*_i._
- the estimated number of deaths due to disease D in year *i* that were prevented by non-vaccine-related factors that were put in place since 198 is *Y*(0) *p_i_* [*d_1980_* - *d*_i_]_._

**A.6. Estimation of vaccine-prevented measles and pertussis deaths using LiST**

The effect sizes and general model for LiST have been developed by the Child Health Epidemiology Reference Group (CHERG) and are being updated regularly to reflect the most up-to-date knowledge available.

Coverage of child-related health interventions (such as antenatal care, delivery care and child treatment) were collated from a variety of sources including Demographic and Health Surveys (1997 and 2002) and Multiple Indicator Cluster Surveys (2000 and 2006). Additional data on demographics were available from the 1994 Intercensal Demographic Survey, via a publication on Maternal Mortality in Studies in Family Planning. Coverage rates for routine and campaign vaccination in 1980-2010 were obtained from NIHE. Stunting and severe wasting rates were available from the national nutritional surveillance dataset. This was supplemented with data from the WHO Nutrition database for earlier years. Annual causes of death for children 0-5 years of age in 2000-2010 were obtained from published estimates from the Child Health Epidemiology Reference Group (CHERG) (Liu et al, 2012). For mortality, Interagency working Group for Mortality Estimation estimates of under 5 mortality and IMR in 1980 were used. WHO estimates of neonatal mortality in 1990 were extrapolated to 1980 using the backwards trend between 1980 and 1990 from the Institute for Health Metrics and Evaluation.

A baseline population projection was created for 1980-2010, including all readily available demographic and HIV status data. A complete LiST projection was then constructed for 2000 using all coverage data, health status information and mortality rates. The 1980 coverage and health status rates were entered for the years 2001-2005 in order to extrapolate the most likely proportionate cause of death in neonates and 1-59 months olds using a method previously reported in the literature (Amouzou et al, 2010). These values were then used in the baseline LiST projection for the years 1980-2010. The causes of death in 1980 were then adjusted to ensure that predicted numbers of deaths due to measles in 2000-2010 were similar to published CHERG estimate (Liu 2010). Finally, the overall mortality rates predicted by the model were compared to those presented by IGME. The neonatal mortality rate reduction was underestimated, likely due to incomplete data on changes in coverage of health interventions in Vietnam prior to 1997 and after 2006. Coverage rates were adjusted for select unmeasured health interventions in the neonatal period until the model produced neonatal and under-five mortality rates which were within the IGME/WHO confidence intervals. The neonatal mortality rate in LiST each year in the period 1980-2010 was set to match the observed mortality rate from national statistics, in order to ensure the correct number of individuals exposed to mortality risks.

**A.7. Details about estimates of EPI costs**

Vaccines are delivered via a network of community health workers, mostly through fixed immunisation days at commune health centres. Data sources used to estimate EPI costs are as follows:

| Data source | Date range | Cost items |
| --- | --- | --- |
| Comprehensive multi-year plan for immunisation [2] | 2011-2015 | Staff allowances, supplies, operating costs (electricity, water, telephone, fax), other recurrent costs (stationery, maintenance) |
| Study on cost of providing the expanded programme on immunization in a rural district of Vietnam [3] | 2005 | Staff allowances for vaccines delivered via community health workers. |
| Study on the logistic costs of immunization [4] | 2010 | Depreciation of capital items (buildings, equipment, vehicles, furniture) |
| National EPI | 2005-2010 | Number of vaccine doses used |

Annual costs by year (in millions of 2010 US$) are as follows:

|  | **1996** | **1997** | **1998** | **1999** | **2000** | **2001** | **2002** | **2003** |
| --- | --- | --- | --- | --- | --- | --- | --- | --- |
| **DTP** | 3.45 | 3.27 | 3.06 | 2.85 | 2.8 | 2.74 | 2.1 | 2.7 |
| **OPV** | 2.24 | 2.12 | 1.99 | 1.85 | 1.82 | 1.78 | 1.67 | 1.79 |
| **MCV1** | 0 | 0 | 0 | 0 | 0 | 0 | 0 | 0 |
| **MCV2** | 0 | 0 | 0 | 0 | 0 | 0 | 0 | 0 |
| **DTP campaign** | 0 | 0 | 0 | 0 | 0 | 0 | 0 | 0 |
| **OPV campaign** | 12.7 | 12.1 | 7.46 | 7.44 | 2.17 | 0.41 | 0.41 | 0.1 |
| **MCV campaign** | 0.08 | 0.02 | 0.02 | 0.26 | 1.27 | 0.23 | 5.38 | 6.19 |
| **Total** | 18.47 | 17.51 | 12.53 | 12.4 | 8.06 | 5.16 | 9.56 | 10.78 |
|  |  |  |  |  |  |  |  |  |
|  | **2004** | **2005** | **2006** | **2007** | **2008** | **2009** | **2010** | **Total** |
| **DTP** | 2.64 | 2.63 | 2.64 | 2.64 | 2.72 | 2.8 | 2.74 | 41.8 |
| **OPV** | 1.83 | 1.82 | 1.83 | 1.83 | 1.88 | 1.95 | 1.89 | 28.3 |
| **MCV1** | 0 | 1.42 | 1.41 | 1.27 | 1.46 | 1.51 | 1.53 | 8.61 |
| **MCV2** | 0 | 0.02 | 1.15 | 0.91 | 0.94 | 1.18 | 1.2 | 5.41 |
| **DTP campaign** | 0.25 | 0 | 0 | 0 | 0 | 0 | 0 | 0.25 |
| **OPV campaign** | 0.54 | 1.65 | 1.89 | 0 | 0 | 0 | 0 | 46.8 |
| **MCV campaign** | 1.28 | 0 | 0 | 2.75 | 0.74 | 0 | 5.19 | 23.4 |
| **Total** | 6.54 | 7.54 | 8.92 | 9.4 | 7.74 | 7.44 | 12.55 | 154.57 |

Reference List

[1] Cleveland,W.S., Grosse E, Shyu MJ. Local regression models. Statistical Models in S.New York, Chapman and Hall, 1992: p. 309-76.

[2] Ministry of Health of Viet Nam. Multi-year Plan for Immunization (cMYP) 2011-2015. Hanoi: National Expanded Program on Immunization.

[3] Hoang MV, Nguyen TB, Kim BG, Dao LH, Nguyen TH, Wright P. Cost of providing the expanded programme on immunization: findings from a facility-based study in Viet Nam, 2005. Bull World Health Organ 2008 Jun;86(6):429-34.

[4] World Health Organization, PATH. Optimize: Vietnam Report. Seattle: PATH, WHO; 2013.
